# Left-sided atrial tachycardia ablation in an atrial-esophageal fistula survivor

**DOI:** 10.1016/j.hrcr.2022.03.008

**Published:** 2022-03-18

**Authors:** Sevasti-Maria Chaldoupi, Bart Maesen, Dominik Linz, Justin G.L. M. Luermans

**Affiliations:** ∗ Department of Cardiology, Maastricht University Medical Center, Maastricht, the Netherlands; † Department of Cardiothoracic Surgery, Maastricht University Medical Center, Maastricht, the Netherlands; ‡ Cardiovascular Research Institute Maastricht (CARIM), Maastricht University, Maastricht, the Netherlands; § Department of Cardiology, Radboud University Medical Center, Nijmegen, the Netherlands

**Keywords:** Atrial fibrillation, Atrial tachycardia, Atrial-esophageal fistula, High-density electroanatomical mapping, Radiofrequency AF ablation, Pulmonary vein isolation, Posterior box isolation

## Introduction

Although atrial-esophageal fistula (AEF) following atrial fibrillation (AF) catheter ablation is a rare complication, occurring in 0.02%–0.11% of cases, it is associated with a high mortality rate.^1^ The proximity^2^ of the esophagus to the posterior wall of the left atrium (LA) is the most important factor for the pathogenesis of esophageal injury. The precise mechanism of esophageal injury is not completely understood; several theories have been suggested, such as mucosal damage of the esophagus owing to thermal injury during ablation or indirectly owing to ischemic injury through thermal occlusion of the end-arterioles.^3^ The delayed clinical manifestation of an AEF at 2–5 weeks after the AF ablation procedure makes an involvement of mechanical perforation of the atrial wall during the procedure unlikely. Early detection of AEF and urgent surgical repair are the cornerstone for successful treatment and management.^1^ Because of the rarity of such a complication and its potentially fatal outcome, until now most case reports and reviews have mainly focused on the clinical presentation, pathogenesis, and management of AEF.^4^, ^5^, ^6^, ^7^ Data on rhythm management and the feasibility of an endocardial redo ablation procedure in patients who have survived an AEF and still experience disabling symptoms owing to recurrent AF episodes or other atrial tachycardia (AT) are sparse. In this case report, we describe a redo AF ablation procedure in a patient after surgical AEF who experienced highly symptomatic recurrences of AF / left-sided AT refractory to pharmacological treatment.

## Case report

A 54-year-old female patient, who had an initial AF ablation procedure complicated by an AEF 2 years ago, was referred with recurrent highly symptomatic AF / left-sided AT despite amiodarone to our center for a second opinion concerning rhythm management and AF ablation redo procedure. She was also known to have hypertrophic cardiomyopathy with a CHA_2_DS_2_-VASc 1 (female) and she was treated with oral anticoagulation. The AF radiofrequency (RF) ablation procedure 2 years before consisted of isolation of all pulmonary veins and a posterior wall (box) isolation. During the procedure, irrigated RF ablation with a maximum energy of 30 W was used on the posterior wall and 35 W on the anterior wall. Three weeks after the initial procedure, the patient presented with cerebrovascular ischemic events owing to air embolisms. She was diagnosed with AEF and underwent emergency surgery. The necrotic zone of 0.5–1 cm of the posterior LA wall was repaired with a bovine pericardial patch. The esophageal injury was closed with a pleural patch. The postoperative period was uneventful and only minor residual neurological symptoms remained.

Under amiodarone treatment the electrocardiogram showed sinus rhythm with a pre-existent right bundle brunch block and multiple AF and AT with high symptom-rhythm correlation. Transthoracic echocardiography showed known left ventricle hypertrophy with an interventricular septum thickness up to 20 mm, preserved left ventricular systolic function, and a significant LA dilatation with LA volume index of 58 mL/m^2^.

A cardiac contrast computed tomography (CT) scan was performed to assess the anatomy of the pulmonary veins and the proximity of the esophagus to the LA. The atrial and esophageal patch could not be visualized on the CT scan. A cardiothoracic surgeon was consulted, and we were advised to avoid RF ablation on the posterior wall because of the risk of suture rupture potentially resulting in perforation and reoccurrence of AEF. The therapeutic options and limitations, including the risks and potential alternative therapies, were discussed with the patient and informed consent for a diagnostic procedure and redo RF ablation avoiding the mid posterior wall was obtained.

**Figure 1:**
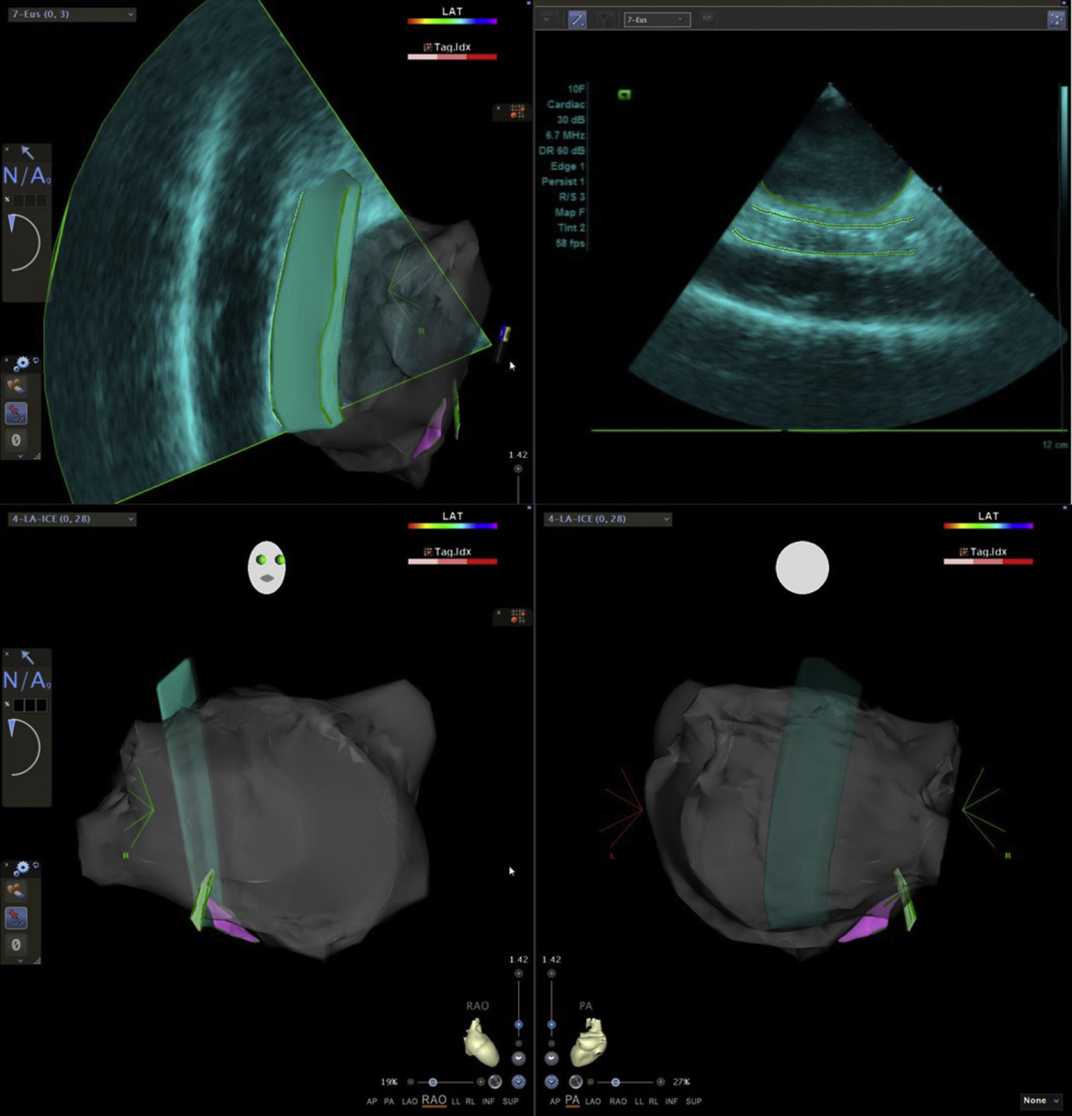
A 3D reconstruction of the left atrium, pulmonary veins, and the esophagus with SoundStar catheter (3D diagnostic ultrasound catheter, 10F, 90 cm; Biosense Webster, Inc, Diamond, Bar, CA) and CartoSound mapping system. The esophagus temperature probe is also visible.

**Figure 2:**
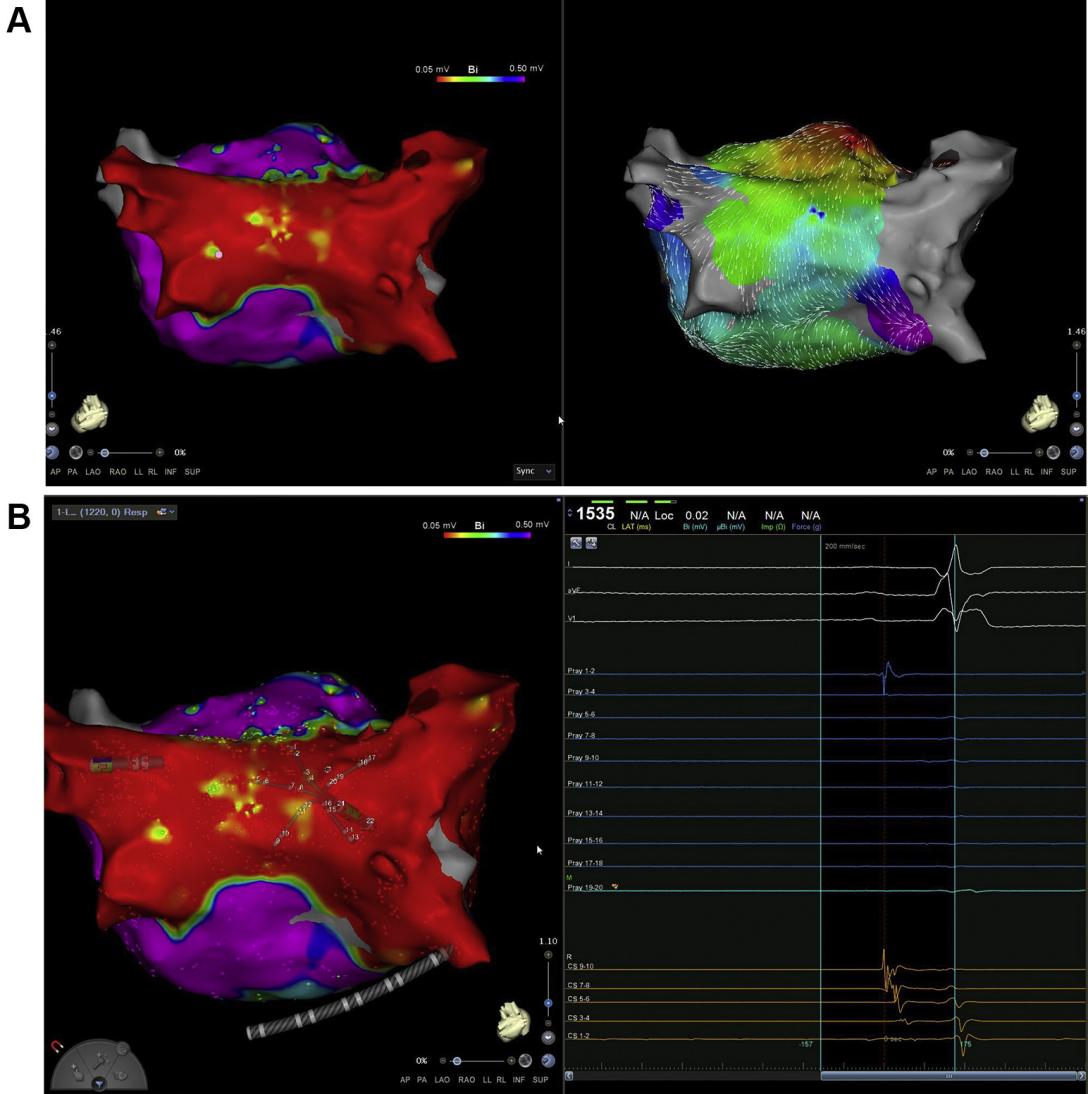
Activation and voltage mapping during sinus rhythm. A: Although the electrograms of the posterior wall are <0.05 mV, there is an obvious activation wavefront going from the roof of the left atrium to the posterior-inferior line, indicating that the roof line is not complete, while in the inferior line there is a wavefront collision. B: On the posterior wall we could only localize a very limited area with no local electrical activity, which could represent the area of the atrial patch.

**Figure 3:**
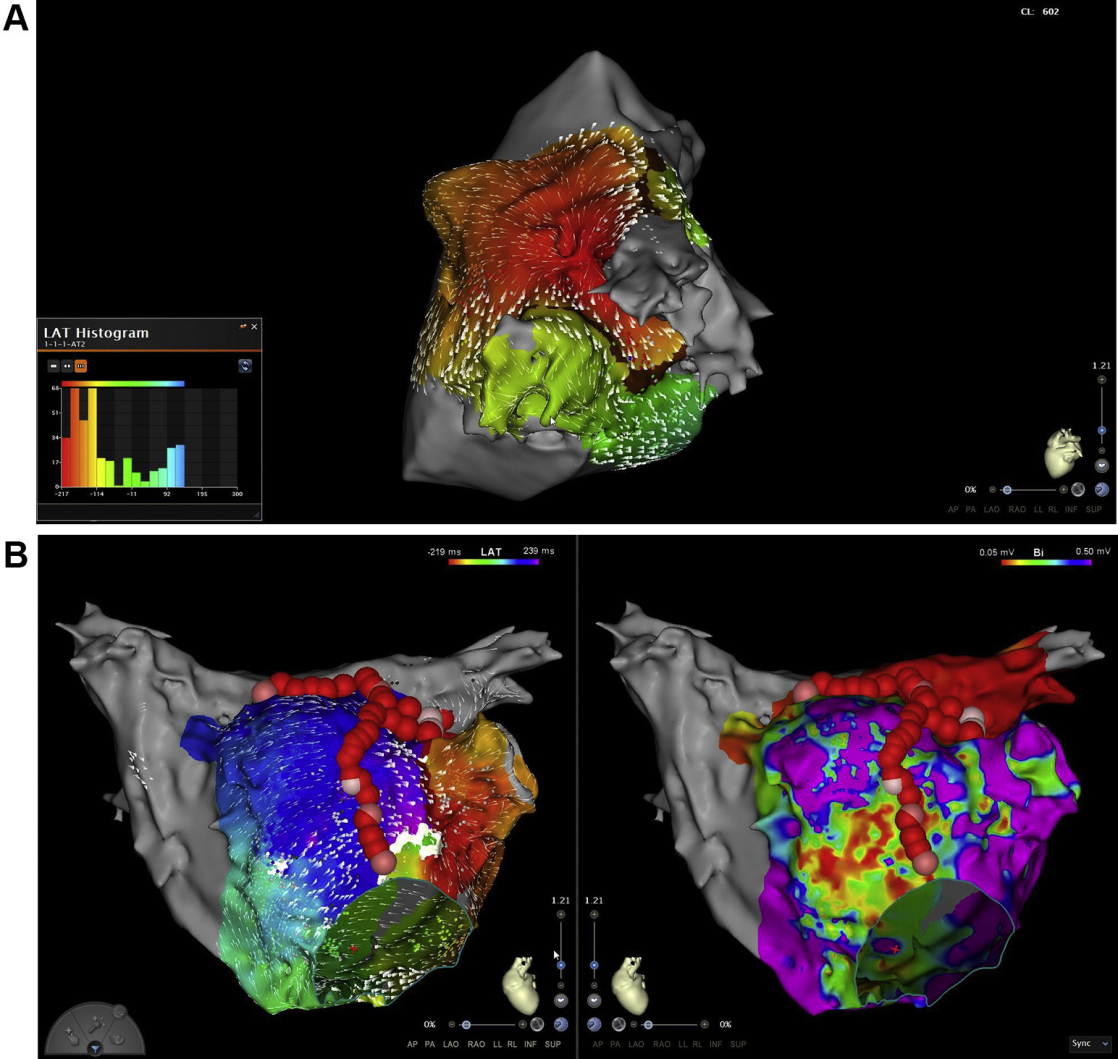
Induced clinical tachycardias. A: Activation mapping of a focal atrial tachycardia originating from the ridge between the left atrial appendage and left upper pulmonary vein. B: Activation and voltage mapping during a macroreentrant tachycardia perimitral in a clockwise direction and anterior line, which terminated the tachycardia (the ablation lesions of the focal tachycardia, anterior roof line, and the anterior line are also visible).

The procedure was performed under general anesthesia and uninterrupted warfarin. A multi-thermocouple temperature probe (SensiTherm; St. Jude Medical, Inc, St. Paul, MN) was used for continuous esophagus temperature monitoring. A right and a left femoral venous access was obtained. A standard decapolar catheter (Webster CS Catheter; Biosense Webster, Inc, Diamond Bar, CA) was placed in the coronary sinus and used for pacing and recording. A 3D intracardiac echocardiography (ICE) image of esophagus and LA with pulmonary veins (PVs) was acquired using the SoundStar catheter (3D diagnostic ultrasound catheter, 10F, 90 cm; Biosense Webster, Inc) and integrated by the CartoSound mapping system.^8^ As expected, the posterior wall of the LA was in close proximity with the esophagus. The esophageal probe but not the patches could be visualized with ICE (Figure 1). Both imaging modalities, the preprocedural CT and per-procedural ICE, were also used to exclude left atrial appendage thrombus prior to ablation in this patient with AF and hypertrophic cardiomyopathy. Afterward, ICE was used to guide transseptal puncture and assist the positioning of the mapping catheter (PentaRay catheter; Biosense Webster, Inc) and the ablation catheter (ThermoCool SmartTouch SF catheter; Biosense Webster, Inc).^9^ On high-density electroanatomical voltage mapping of the LA during sinus rhythm (CARTO 3 V6; Biosense Webster, Inc), all PVs were isolated, but the posterior wall was not isolated owing to a gap in the roof line (Figure 2A). During the electroanatomical mapping of the posterior wall we identified a small area without electrical activity, which might represent the location of the atrial patch (Figure 2B). The remaining posterior box showed low-voltage, fractionated local electrograms and a conduction wavefront from the roof line toward the inferior line, indicating that it was not isolated. Interestingly enough, the surrounding atrial myocardial tissue demonstrated high-voltage areas (Figure 2B). The most common clinical AT was induced repeatedly by catheter manipulation. Activation mapping revealed a focal AT on the ridge between the basis of the left atrial appendage and the anterior side of the ostium of the left upper PV (Figure 3A). After RF ablation with maximal energy of 30 W in this area, the tachycardia terminated. Subsequently we created a new roof line more anteriorly with a maximal energy of 25 W, starting from the left upper PV (at the area where RF ablation of the clinical AT was performed) until the right upper PV, resulting in reisolation of the posterior wall and demonstrable bidirectional block. Afterward, using programmed stimulation, a second AT was induced with an eccentric atrial activation seen in the coronary sinus catheter. Activation mapping revealed a clockwise perimitral atrial flutter. An anterior line from the anterior roof line to the mitral valve annulus was created with maximal energy of 30 W, which terminated the tachycardia (Figure 3B). A bidirectional block afterward was also confirmed. No further sustained AT was inducible by programmed atrial stimulation before and after isoprenaline infusion. Finally, we also performed a cavotricuspid isthmus line, as a typical atrial flutter could not be ruled out based on her clinically documented arrhythmias.

The peri- and postoperative course was uneventful. The day after the procedure, the patient was discharged and amiodarone was discontinued. A 24-hour rhythm monitoring at 4 weeks after the procedure revealed sinus rhythm without any atrial tachyarrhythmias. During the follow-up appointments at 3–6 and 12 months, the patient did not report any symptoms.

## Discussion

To the best of our knowledge, this is the first case of a patient who underwent a redo LA RF ablation procedure for symptomatic and disabling drug-refractory AF and left-sided AT after surgical repair of an AEF. Patients surviving an AEF still have a 30%–40% chance of recurrence of their arrhythmia after their initial AF procedure.^4^

This case shows that a redo RF procedure in these patients is feasible and can be performed safely with satisfactory clinical outcomes, but there are also some limitations. Our approach includes the use of ICE in order to continuously visualize and monitor the position of the esophagus during the procedure and to guide transseptal puncture; for obvious reasons, a transesophageal echocardiogram should be avoided after surgical repair of an AEF. Of note, neither on CT angiography nor on ICE could the patch of the LA posterior wall be visualized. Therefore, we relied on activation mapping to localize the patch, as it is electrically silent. Fortunately, all PVs were isolated in our case, and therefore we could avoid RF ablation at the posterior LA or near the atrial patch. However, if the PVs would have been reconnected, a more distal reisolation of the PVs or a single ring isolation with inferior line sparing could have been a bail-out option.^10^^,^^11^ We identified a gap in the roof line and the posterior wall could be reisolated by RF ablation anterior to the initial roof line to avoid ablation on the posterior wall. The clinical arrhythmias in this patient were a left-sided focal AT and a macroreentrant AT around the mitral valve. This is in accordance with earlier descriptions indicating that 2.8%–11% of the patients undergoing a circumferential antral PV isolation develop AT owing to macroreentry or a focal mechanism.^12^^,^^13^ Fortunately, we did not encounter arrhythmias originating from the posterior atrial patch in this case. Although the arrhythmias in our patient had to be induced, their cycle lengths matched those of the clinically documented ATs and no other arrhythmias could be induced after their elimination. The delivery of maximal energy at these areas remote from the previous scene of calamity was not compromised, ensuring transmurality of our ablation applications. However, during application of the anterior new roof line the energy settings were adjusted to lower limits for the close anatomical proximity of the esophagus to the posterior wall and the fact that we could not be certain of the precise anatomical extension of the atrial patch and the thickness of its surrounding atrial wall.

With this case we demonstrate for the first time the endocardial LA electroanatomical aspect during sinus rhythm after surgical repair of an AEF; the arrhythmias related to such a complication, beyond recurrence of AF; and a stepwise strategy toward efficient and safe treatment of those patients.

## Conclusion

We present a case with favorable outcome of a patient suffering from drug-refractory symptomatic AF and left-sided ATs after surviving a surgical repair of an AEF secondary to radiofrequency AF ablation in which a redo RF ablation in the LA was performed. This is the first case to demonstrate that returning to the “scene of calamity” and RF ablation can be considered if the patient’s symptoms are caused by atrial arrhythmias and if the atrial arrhythmias originate away from the posterior wall. However, caution is warranted, and patients should be selected carefully and be thoroughly informed about the limitations, success rates, and potential risks of such a procedure.

**Key teaching points**

• Redo ablation and rhythm management in patients who have survived an atrial-esophageal fistula and who have recurrent episodes of atrial fibrillation or other left-sided atrial tachycardias is feasible and can be performed safely, with satisfactory clinical results.
• The recurrent atrial arrhythmias in patients surviving an atrial-esophageal fistula may be outside the “scene of the calamity” and may involve left-sided atrial tachycardias owing to macroreentry and/or a focal mechanism.
• Efficient and safe treatment of those patients requires careful patient selection and a stepwise strategy.
